# Differentiating high‐ and low‐grade serous ovarian carcinoma using radiomics: a pilot study

**DOI:** 10.1002/uog.70331

**Published:** 2026-09-13

**Authors:** F. Ciccarone, G. Zinicola, E. H. Tran, G. Baldassari, E. Boccia, C. Nero, F. Moro, T. Pasciuto, G. Scambia, G. Ferrandina, A. Fagotti, A. C. Testa, D. Lorusso, Collaborators, F. Borella, F. Buonomo, R. Fruscio, F. Pozzati, R. Mancari

**Affiliations:** ^1^ Department of Woman and Child Health Fondazione Policlinico Universitario ‘A. Gemelli’ IRCCS Rome Italy; ^2^ Radiomics G‐STeP Research Core Facility, Fondazione Policlinico Universitario ‘A. Gemelli’ IRCCS Rome Italy; ^3^ Section of Obstetrics and Gynecology University Department of Life Sciences and Public Health, Università Cattolica del Sacro Cuore Rome Italy; ^4^ UniCamillus Saint Camillus International University of Health Sciences Rome Italy; ^5^ G‐AURIG – Gemelli Advanced Ultrasound Research and Innovation Group, Fondazione Policlinico Universitario ‘A. Gemelli’ IRCCS Rome Italy; ^6^ Research core facility Data Collection G‐STeP Fondazione Policlinico Universitario ‘A. Gemelli’ IRCCS Rome Italy; ^7^ Section of Hygiene University Department of Life Sciences and Public Health, Universitá Cattolica Del Sacro Cuore Rome Italy; ^8^ Sciences Humanitas University Milan Italy; ^9^ Humanitas San Pio X Milan Italy

**Keywords:** high‐grade serous ovarian cancer, low‐grade serous ovarian cancer, radiomics, ultrasonography

## Abstract

**Objective:**

To identify ultrasound‐based radiomics features capable of distinguishing between high‐grade serous ovarian carcinomas (HGSC) and invasive low‐grade serous ovarian carcinomas (LGSC), and to develop machine‐learning models that include radiomics features to discriminate between the two.

**Methods:**

This was a single‐center, analytical, observational, retrospective pilot study of patients referred to Fondazione Policlinico Universitario ‘A. Gemelli’ IRCCS, Rome, Italy, between January 2014 and June 2022 with a histological diagnosis of HGSC or invasive LGSC, who had DICOM images available from a preoperative ultrasound examination. The extracted radiomics features belonged to two different families: intensity‐based statistical features and textural features. Feature selection was performed via univariate analysis using the Wilcoxon–Mann–Whitney statistical test, followed by multivariate selection using the Boruta algorithm, Pearson correlation analysis to reduce collinearity and Akaike information criterion stepwise regression. Selected features were used to train logistic regression models. To distinguish between HGSC and LGSC, two logistic regression models were developed: a radiomics model based solely on radiomics features, and a clinical–radiomics model combining radiomics features with patient age. For comparison, two additional models were constructed: a radiomics–ultrasound model incorporating radiomics features and ultrasound variables that were significantly different between the two histotypes, and an ultrasound model based exclusively on these ultrasound variables. The area under the receiver‐operating‐characteristics (ROC) curve (ROC‐AUC) and the area under the precision‐recall (PR) curve (PR‐AUC) were calculated. Modeling results were internally validated via the bootstrap resampling method, and optimism‐corrected metrics were computed.

**Results:**

A total of 153 patients (120 with a histological diagnosis of HGSC and 33 with a histological diagnosis of invasive LGSC) were recruited, providing a total of 332 images for analysis. For each image, 75 radiomics features were extracted. After feature selection, three textural features, ‘gray‐level co‐occurrence matrix informational measure of correlation 2’ (‘F_cm.info.corr.2’), ‘gray‐level run length matrix run percentage’ (‘F_rlm.r.perc’) and ‘gray‐level size zone matrix zone size variance’ (‘F_szm.zs.var’) were used for the radiomics model; one textural feature (F_szm.zs.var) and age were used for the clinical–radiomics model; one textural feature (F_cm.info.corr.2) and one ultrasound variable (color score) were used for the radiomics–ultrasound model; and three ultrasound variables (color score, presence of papillary projections, presence of the ovarian crescent sign) were used for the ultrasound model. The internally validated (optimism‐corrected) ROC‐AUC was 0.69 (95% CI, 0.57–0.77) for the radiomics model, 0.80 (95% CI, 0.72–0.90) for the clinical–radiomics model, 0.68 (95% CI, 0.56–0.77) for the radiomics–ultrasound model and 0.78 (95% CI, 0.68–0.88) for the ultrasound model. The clinical–radiomics model performed similarly to the ultrasound model (optimism‐corrected ROC‐AUC, 0.80 *vs* 0.78; *P* = 0.726) and better than the radiomics (optimism‐corrected ROC‐AUC, 0.80 *vs* 0.69; *P* = 0.042) and radiomics–ultrasound (optimism‐ corrected ROC‐AUC, 0.80 *vs* 0.68; *P* = 0.048) models.

**Conclusions:**

Our findings indicate that, despite detectable differences in radiomics features between HGSC and LGSC, current radiomics and imaging‐based approaches do not provide sufficient added value over conventional ultrasound for reliable preoperative discrimination. Future research may be necessary to develop more accurate and clinically useful artificial intelligence‐based predictive models. © 2026 The Author(s). *Ultrasound in Obstetrics & Gynecology* published by John Wiley & Sons Ltd on behalf of International Society of Ultrasound in Obstetrics and Gynecology.

## INTRODUCTION

Ovarian cancer is the second most common gynecological malignancy in developed countries and the third in developing countries^1^. The majority of ovarian malignancies (95%) are epithelial^2^, and serous carcinomas account for 80% of epithelial ovarian cancers. However, high‐grade serous ovarian carcinomas (HGSC) and low‐grade serous ovarian carcinomas (LGSC) have different pathogenesis, response to chemotherapy and prognosis^3^. HGSC comprise 75% of all serous ovarian malignancies, typically presenting at an advanced stage^2^, with a mean age of 55–65 years at diagnosis^4^. HGSC typically show strong positivity for p53, WT1, p16 and BRCA1, and expresses estrogen‐progestogen receptors less frequently^2^, ^3^, ^4^, ^5^. LGSC account for less than 5% of all epithelial ovarian cancer^2^, ^3^, ^4^, ^5^. Patients with LGSC are younger than those with HGSC, with a mean age at diagnosis of 45 years for non‐invasive and 53 years for invasive LGSC^4^. LGSC is associated not with *BRCA* mutations, but instead with *BRAF*, *KRAS* and *ERBB2* mutations^6^, ^7^. LGSC are diffusely positive for CK7, PAX8, ER, PR and WT1 proteins, with patchy p16 expression and wild‐type p53 patterns^8^.

Patients affected by serous carcinoma can be asymptomatic or report early satiety, bloating, dyspnea, urinary urgency and pain. In advanced cases, pleural effusion and bowel obstruction may occur^9^. LGSC are associated with higher 5‐year progression‐free survival and overall survival than HGSC, while at 10 years the prognoses overlap^10^.

Complete cytoreduction (residual tumor = 0) is the goal of treatment, although a tumor residue of < 1 cm is still considered optimal cytoreduction in both HGSC and LGSC^11^. However, since LGSC are less chemosensitive, the maximum surgical effort in these cases is mandatory. The therapeutic landscape for HGSC is considerably broader than that for LGSC. Standard first‐line treatment includes cytoreductive surgery and platinum‐based chemotherapy, while targeted therapy with bevacizumab and maintenance therapy with poly(ADP‐ribose) polymerase (PARP) inhibitors are available for selected patients, according to disease characteristics and *BRCA* mutation and homologous recombination deficiency status. Furthermore, immunotherapy has shown clinical benefit in specific subgroups of platinum‐resistant ovarian cancer. Such differences highlight the importance of accurately distinguishing HGSC from LGSC, as the two diseases require fundamentally different therapeutic approaches^12^. Ultrasound is considered the first‐line imaging method for diagnosing ovarian masses, offering high accuracy in distinguishing between benign and malignant lesions. Radiomics is an emerging field that extracts high‐dimensional data from radiological images to describe tissue characteristics, shape and texture^13^, ^14^, conveying meaningful information about tumor biology, behavior and pathophysiology^15^. We hypothesized that radiomics features could enhance the preoperative diagnosis of ovarian masses by reflecting histopathological, genetic and clinical heterogeneity through different imaging phenotypes. The aim of this study was to identify ultrasound‐based radiomics features capable of distinguishing between HGSC and invasive LGSC, and to develop machine‐learning models that include radiomics features to discriminate between the two.

## METHODS

### Study design

This was a single‐center, analytical, observational, retrospective pilot study developed and performed by the Multicenter Italian Trials in Ovarian Cancer and Gynecologic Malignancies (MITO) group. All procedures were conducted in accordance with the principles outlined in the Declaration of Helsinki, and ethical approval was obtained from the local research ethics committee of Fondazione Policlinico Universitario ‘A. Gemelli’ IRCCS, Rome, Italy (Protocol ID: 3963).

All patients with a histological diagnosis of HGSC or invasive LGSC referred to the Fondazione Policlinico Universitario ‘A. Gemelli’ IRCCS between January 2014 and June 2022 were identified from the Gynecological Oncology Unit database. Patients were eligible for inclusion if they met the following criteria: (1) at least 18 years of age; (2) histological diagnosis of HGSC or invasive LGSC; (3) preoperative ultrasound examination performed at our institution before the initial diagnosis; and (4) availability of preoperative ultrasound images in DICOM format. Patients were excluded from the study if they: (1) did not have preoperative ultrasound images in DICOM format or had poor‐quality DICOM images that prevented visualization and delineation of the ovarian mass, or if the images contained calipers, text or a color Doppler box; (2) did not have preoperative baseline ultrasound images acquired before neoadjuvant chemotherapy (which may modify tumor characteristics, including morphology and vascularization, and consequently affect radiomics features); or (3) had experienced a pathological recurrence. Some of the selected patients had previously been included in ongoing prospective observational International Ovarian Tumor Analysis (IOTA) studies (IOTA phase 5^16^ and IOTA phase 7; the latter is registered at ClinicalTrials.gov (NCT02847832)).

Histological diagnosis and FIGO (International Federation of Gynecology and Obstetrics) stage^17^ were obtained from the patients' medical records. All ultrasound examinations were performed at our institution, and lesion ultrasound descriptors were assigned prospectively at the time of the scan. Radiomics and data analysis were both carried out retrospectively at Fondazione Policlinico ‘A. Gemelli’ IRCCS of Rome.

### Ultrasound imaging

Transvaginal and transabdominal pelvic ultrasound examinations were performed within 180 days before surgery. All ultrasound examinations were performed by an experienced examiner (Level II according to the European Federation of Ultrasound Societies in Medicine and Biology (EFSUMB)) with high‐level ultrasound equipment from different vendors^18^: Voluson E10 (GE Healthcare, Zipf, Austria), Samsung W10, HERA I10, HS70A or WS80A (Samsung Medison Co., Ltd, Seoul, Republic of Korea), Canon Aplio series (Canon Medical Systems Corp., Otawara, Japan) or Esaote system (Esaote S.p.A., Genoa, Italy). The frequency of the transvaginal probes varied between 5.0 and 9.0 MHz and that of the tansabdominal probes varied between 3.5 and 5.0 MHz. All ultrasound images were acquired according to the current standardized examination technique^15^. All ovarian masses were described using the terminology and definitions established by the IOTA group^19^. In case of patients with bilateral ovarian masses, only the dominant mass was analyzed. When more than one image had been acquired for the dominant mass (images obtained from different scan planes or multiple images obtained in order to capture the morphological complexity of the ultrasound image), all were used for radiomics analysis; the maximum number of images available for a single case was five.

### Image postprocessing and segmentation

At the Fondazione Policlinico Universitario ‘A. Gemelli’ IRCCS, transabdominal and transvaginal grayscale ultrasound images of adnexal masses were stored on external hard disks until 2019, and then in a picture archiving and communication system. Images in DICOM (.dcm) format were collected retrospectively for subsequent analysis in this study. All the image files had been anonymized previously, according to current privacy regulations^20^.

All ultrasound images were converted from red, green and blue format to grayscale and subsequently segmented to define the region‐of‐interest (ROI) (Figure 1). The ROI in each image was manually segmented by a single trained gynecologist (G.Z.) with EFSUMB Level‐II experience of ultrasound in gynecology, using the software Aliza Medical Imaging & DICOM Viewer, version 2.3.10 (Aliza Medical Imaging, Bonn, Germany). The ROI was delineated including only the solid component of the mass. In case of an adnexal mass with cystic areas, the examiner excluded from the ROI any cyst with a diameter > 5 mm (Figure 1), because we wanted to analyze the ultrasound characteristics of just the solid tissue.

**Figure 1:**
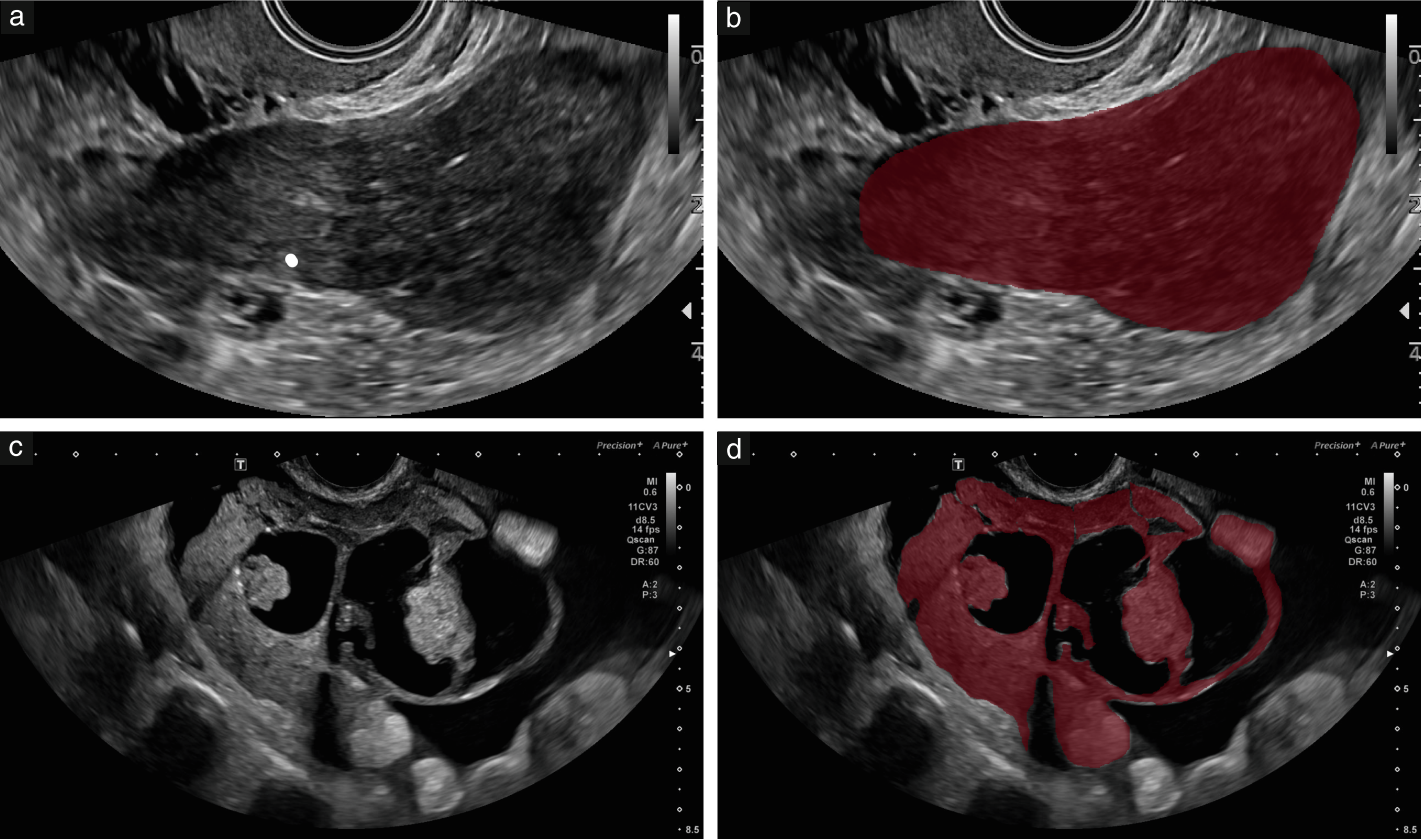
Examples of segmentation showing grayscale ultrasound images (a,c) and grayscale ultrasound images with region‐of‐interest mask applied (b,d). (a,b) High‐grade serous ovarian carcinoma, with solid appearance at ultrasound examination. (c,d) Low‐grade serous ovarian carcinoma, with multilocular‐solid appearance at ultrasound examination.

### Extraction of radiomics features

Radiomics features were extracted from ROIs using MODDICOM, an open‐source R library implemented by the Radiomics Research Core facility of the Fondazione Policlinico Universitario ‘A. Gemelli’ IRCCS^21^. Feature extraction was fully compliant with the Image Biomarker Standardisation Initiative (IBSI) guidelines^22^. The following feature families were extracted: intensity‐based statistical features (F_stat), textural features based on the gray‐level co‐occurrence matrix (F_cm), textural features based on the gray‐level size zone matrix (F_szm) and textural features based on the gray‐level run‐length matrix (F_rlm). Intensity‐based statistical features are related to the statistical properties of the gray‐level histogram within the ROI and provide quantitative measures on a global scale, while textural features describe the local distribution of the gray levels within the ROI. For patients who had more than one image available, feature values were averaged over the images to avoid redundancy and bias during model training.

### Statistical analysis and radiomics modeling

Categorical clinical, ultrasound and histological data are reported as *n* (%), while continuous variables are summarized as mean ± SD.

Four machine‐learning models were developed to differentiate between HGSC and LGSC: a radiomics model, based exclusively on radiomics features; a clinical–radiomics model, incorporating the patient's age and radiomics features; a radiomics–ultrasound model, integrating ultrasound variables with radiomics features; and an ultrasound model, constructed solely from ultrasound variables.

For development of the radiomics model, feature selection was performed initially through univariate analysis using the Wilcoxon–Mann–Whitney test, with a *P*‐value < 0.05 considered statistically significant, followed by multivariate selection with the Boruta algorithm^23^. To further reduce dimensionality and mitigate redundancy arising from linear correlations among features, pairwise Pearson correlation coefficients were computed and a threshold of 0.7 was used to exclude correlation between variables: for each pair of features with an absolute correlation > 0.70, the feature with the higher mean absolute correlation with the remaining features was removed. Mean absolute correlations were recalculated after each removal, thereby accounting for the changing correlation structure and avoiding dependence on a fixed order of variable removal. The same feature selection pipeline was applied to build the clinical–radiomics model, using the combined dataset of radiomics features and patient age. However, in this case, the Pearson correlation threshold was set to 0.6 in order to reach a number of variables fulfilling the ‘one‐in‐ten’ rule to ensure a minimum of 10 events per predictor variable included in the logistic regression models and avoiding overfitting. For the ultrasound model, ultrasound variables were selected using either the chi‐square test or Fisher's exact test, as appropriate, with a *P*‐value of 0.05 used as the cut‐off for feature inclusion. For the radiomics–ultrasound model, radiomics features were initially selected using the Wilcoxon–Mann–Whitney test (*P* < 0.05), while ultrasound variables were assessed using the chi‐square test or Fisher's exact test, as appropriate. The selected radiomics and candidate ultrasound variables were then jointly entered into the Boruta algorithm. Highly correlated radiomics features were removed using an absolute pairwise Pearson correlation cut‐off of 0.7. Point‐biserial correlation was employed to assess associations between continuous radiomics features and binary ultrasound variables identified via Fisher's exact test or the chi‐square test, while the Kruskal–Wallis test was used to explore associations between continuous radiomics features and non‐binary discrete ultrasound variables.

For each model, logistic regression was performed with or without an additional stepwise feature selection procedure based on the Akaike information criterion (AIC) to prevent overfitting and following the ‘one‐in‐ten’ rule. Model performance was evaluated in terms of discrimination. We computed the area under the receiver‐operating‐characteristics (ROC) curve (AUC), with 95% CIs calculated via the percentile bootstrap method^24^. The model's performance metrics based on the confusion matrix were computed by using the ‘best threshold’ (i.e. the threshold that provided the largest number of correct classifications according to Youden's index^25^). These metrics included accuracy, sensitivity and specificity, all reported with 95% CI calculated using the Jeffreys method^26^.

Owing to the moderate class imbalance of the dataset (22% LGSC *vs* 78% HGSC), for all the models we also computed the AUC of the precision‐recall (PR) curve, with 95% CIs calculated via the bootstrap method, and compared the two evaluation approaches (ROC‐AUC *vs* PR‐AUC)^27^. The PR curve is more appropriate for imbalanced datasets because it focuses on precision (positive predictive value) and recall (sensitivity), rather than true negatives, which dominate in such cases. As shown by Ozenne *et al*.^27^, the ROC‐AUC can overestimate performance when prevalence is low, while the PR‐AUC better reflects the model's ability to identify true positives.

Modeling results were internally validated via the bootstrap resampling method, with 1000 iterations performed on data according to the TRIPOD guidelines^28^. During each iteration, a logistic regression model was implemented on the bootstrap sample and optimism‐corrected metrics were computed. This process was performed for each developed model. These optimism‐corrected metrics were reported with 95% CIs, calculated using the percentile method, performed by taking the 2.5^th^ and 97.5^th^ percentiles of the optimism‐corrected values. The statistical significance of the difference in optimism‐corrected AUCs between the clinical–radiomics model and each comparison model (radiomics, ultrasound and radiomics–ultrasound) was assessed using empirical two‐sided *P*‐values. These were computed as twice the smaller tail probability from the bootstrap distribution of AUC differences, without assuming any specific direction of superiority. This non‐parametric approach avoids assumptions about the distribution of AUC differences and follows standard bootstrap methodology for hypothesis testing^29^, ^30^.

## RESULTS

### Study population

A total of 2647 patients with a histological diagnosis of HGSC or invasive LGSC referred to our institute were screened for eligibility. After exclusion of 2494 patients owing to a lack of suitable preoperative baseline ultrasound images acquired before the initiation of neoadjuvant chemotherapy, or to disease recurrence, absence of a visible adnexal mass, poor‐quality ultrasound images or a lack of images in DICOM format, 153 patients formed the study cohort and were included in the final analysis: 120 with HGSC and 33 with invasive LGSC (Figure 2).

**Figure 2:**
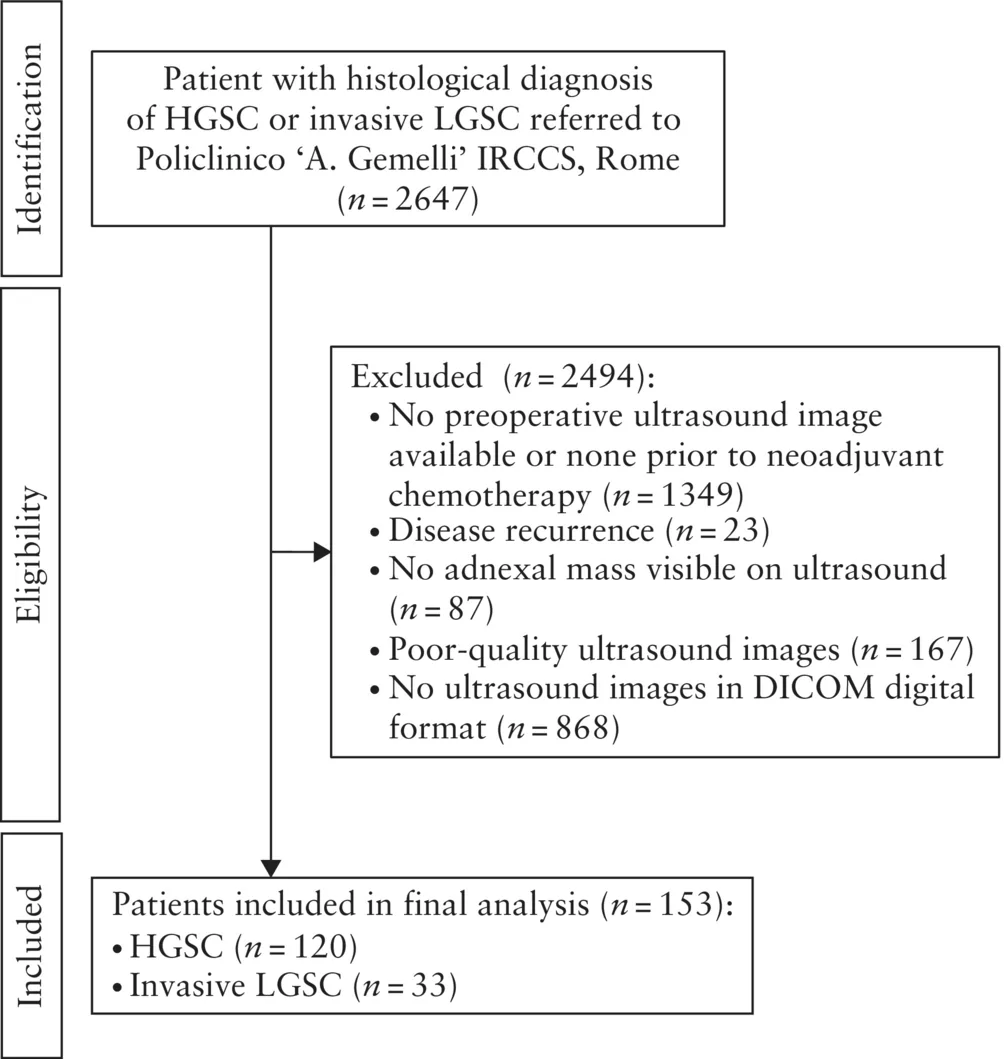
Flowchart summarizing inclusion of patients in study population. HGSC, high‐grade serous ovarian carcinoma; LGSC, low‐grade serous ovarian carcinoma.

For some patients, more than one ultrasound image of the dominant mass was available, with a median of 2 (IQR, 1–3) images per patient. In these cases, up to five images per mass were analyzed, giving a total of 332 images analyzed. Most images were obtained transvaginally (322/332 (97%)), while 10/332 (3%) were obtained transabdominally. The distribution of ultrasound equipment used for examinations was as follows: GE Voluson E10 (*n* = 80 patients), Samsung W10 (*n* = 14 patients), Samsung HERA I10 (*n* = 29 patients), Samsung HS70A (*n* = 7 patients), Samsung WS80A (*n* = 18 patients), Canon Aplio series (*n* = 4 patients) and Esaote system (*n* = 1 patient).

### Clinical, ultrasound and histopathological data

Clinical and histopathological characteristics of patients, both overall and of the HGSC and LGSC groups, are summarized in Table 1. Patients diagnosed with HGSC were older than those with LGSC, with a mean age of 59.7 years *vs* 47.5 years (*P* < 0.0001). A higher proportion of HGSC patients were postmenopausal compared with LGSC patients (93/120 (77.5%) *vs* 10/33 (30.3%); *P* < 0.0001). CA 125 levels were significantly higher in patients with HGSC than in those with LGSC (*P* = 0.008).

**Table 1:** Clinical, molecular and pathological characteristics in 153 women with high‐grade serous ovarian carcinoma (HGSC) or low‐grade serous ovarian carcinoma (LGSC)

Characteristic	All cases (*n* = 153)	HGSC (*n* = 120)	LGSC (*n* = 33)	*P*
Age at diagnosis (years)	57.0 ± 12.3	59.7 ± 10.6	47.5 ± 13.3	< 0.0001
BMI (kg/m^2^)	24.7 ± 4.4	24.5 ± 4.2	25.1 ± 5.2	0.509
Postmenopausal	103 (67.3)	93 (77.5)	10 (30.3)	< 0.0001
Postmenopausal bleeding	8/103 (7.8)	7/93 (7.5)	1/10 (10.0)	0.781
Symptoms				0.056
Asymptomatic	57 (37.3)	40 (33.3)	17 (51.5)	
Symptomatic	96 (62.7)	80 (66.7)	16 (48.5)	
Abdominal and/or pelvic pain	47/96 (49.0)	37/80 (46.3)	10/16 (62.5)	0.253
Abdominal distension	43/96 (44.8)	37/80 (46.3)	6/16 (37.5)	0.521
Increased abdominal size	42/96 (43.8)	36/80 (45.0)	6/16 (37.5)	0.581
Constipation	6/96 (6.3)	6/80 (7.5)	0/16 (0)	0.258
Irregular vaginal bleeding	6/96 (6.3)	4/80 (5.0)	2/16 (12.5)	0.261
Unintentional weight loss	2/96 (2.1)	1/80 (1.3)	1/16 (6.3)	0.386
Urinary frequency	1/96 (1.0)	1/80 (1.3)	0/16 (0)	1
Urinary urgency	1/96 (1.0)	1/80 (1.3)	0/16 (0)	1
Nausea or vomiting	1/96 (1.0)	1/80 (1.3)	0/16 (0)	1
Fatigue	1/96 (1.0)	1/80 (1.3)	0/16 (0)	1
Personal history of cancer				0.611
None	141/148 (95.3)	112/117 (95.7)	29/31 (93.5)	
Breast cancer	7/148 (4.7)	5/117 (4.3)	2/31 (6.5)	
Family history of cancer				0.050
None	65/142 (45.8)	46/111 (41.4)	19/31 (61.3)	
Any cancer	77/142 (54.2)	65/111 (58.6)	12/31 (38.7)	0.443
Ovarian cancer	11/77 (14.3)	9/65 (13.8)	2/12 (16.7)	
Breast cancer	24/77 (31.2)	19/65 (29.2)	5/12 (41.7)	
Endometrial cancer	2/77 (2.6)	2/65 (3.1)	0/12 (0)	
Gastrointestinal cancer	11/77 (14.3)	8/65 (12.3)	3/12 (25.0)	
Other cancer	29/77 (37.7)	27/65 (41.5)	2/12 (16.7)	
CA 125 (IU/mL)*	848 ± 1987.8	993.4 ± 2222.2	328.2 ± 323.9	0.008
*BRCA 1* or *BRCA 2*				0.243
Wild type	56/101 (55.4)	45/85 (52.9)	11/16 (68.8)	
Mutated	45/101 (44.6)	40/85 (47.1)	5/16 (31.3)	0.173
*BRCA1*	25/45 (55.6)	24/40 (60.0)	1/5 (20.0)	
*BRCA2*	11/45 (24.4)	9/40 (22.5)	2/5 (40.0)	
Unknown type of mutation	9/45 (20.0)	7/40 (17.5)	2/5 (40.0)	
p53				< 0.0001
Wild type	25/120 (20.8)	4/99 (4.0)	21/21 (100)	
Mutated	95/120 (79.2)	95/99 (96.0)	0/21 (0)	
FIGO stage				0.574
I	21 (13.7)	15 (12.5)	6 (18.2)	
II	13 (8.5)	10 (8.3)	3 (9.1)	
III	94 (61.4)	73 (60.8)	21 (63.6)	
IV	25 (16.3)	22 (18.3)	3 (9.1)	

Data are presented as mean ± SD, *n* (%) or *n*/*N* (%), where *N* is the number of participants with available data for each variable. Comparisons between HGSC and LGSC by two‐sided Pearson's chi‐square test or Fischer's exact test and Student's *t*‐test, as appropriate. *P* < 0.05 considered statistically significant.

* Data available for 133 patients. BMI, body mass index; *BRCA*, BReast CAncer gene; CA, cancer antigen; FIGO, International Federation of Gynecology and Obstetrics^17^.

The genetic profile of patients in each group is shown in Table 1. Eight‐five of the 120 HGSC patients were tested for mutation of the *BRCA* genes: 45/85 (52.9%) were wild type and 40/85 (47.1%) were mutated. Sixteen of the 33 LGSC patients were tested for *BRCA* gene mutations: 11/16 (68.8%) were wild type and 5/16 (31.3%) were mutated, but this difference between the two groups was not statistically significant. Tumor suppressor p53 was mutated in 95/99 (96.0%) cases of HGSC, and wild type in all 21 cases of LGSC that were tested (*P* < 0.0001). In both groups, most tumors (> 60%) were diagnosed as FIGO Stage III.

The ultrasound characteristics of HGSC and LGSC are summarized in Table 2. The two tumor types exhibited similar morphology, with most masses appearing solid or multilocular‐solid. However, the two groups differed significantly in the presence of papillary projections, hyperechogenic foci, color score and the presence of the ovarian crescent sign. Papillary projections were observed less frequently in HGSC than in LGSC (12/120 (10.0%) *vs* 12/33 (36.4%); *P* < 0.0001). Hyperechogenic foci were observed in 21/33 (63.6%) cases of LGSC and in none of the HGSC cases (*P* < 0.0001). Similarly, the ovarian crescent sign was identified less often in HGSC than in LGSC (4/119 (3.4%) *vs* 6/32 (18.8%); *P* = 0.002). In terms of vascularization, HGSC mostly demonstrated moderate‐to‐abundant blood flow, whereas LGSC was more frequently associated with poor‐to‐moderate vascularization (*P* = 0.004).

**Table 2:** Ultrasound characteristics in 153 women with high‐grade serous ovarian carcinoma (HGSC) or low‐grade serous ovarian carcinoma (LGSC)

Characteristic	All cases (*n* = 153)	HGSC (*n* = 120)	LGSC (*n* = 33)	*P*
Laterality				0.3
Unilateral	117 (76.5)	94 (78.3)	23 (69.7)	
Bilateral	36 (23.5)	26 (21.7)	10 (30.3)	
Largest tumor diameter (mm)*	94.2 ± 49.6	93.4 ± 46.2	97.2 ± 61.0	0.771
Morphology				0.140
Unilocular	1 (0.7)	0 (0)	1 (3.0)	
Unilocular‐solid	6 (3.9)	4 (3.3)	2 (6.1)	
Multilocular	3 (2.0)	2 (1.7)	1 (3.0)	
Multilocular‐solid	51 (33.3)	37 (30.8)	14 (42.4)	
Solid	92 (60.1)	77 (64.2)	15 (45.5)	
Number of locules†				0.857
< 5	4/34 (11.8)	2/22 (9.1)	2/12 (16.7)	
5–9	6/34 (17.6)	4/22 (18.2)	2/12 (16.7)	
≥ 10	24/34 (70.6)	16/22 (72.7)	8/12 (66.7)	
Maximum diameter of largest solid component (mm)	75.7 ± 57.5	78.6 ± 61.0	64.8 ± 40.5	0.236
Presence of papillary projection(s)	24 (15.7)	12 (10.0)	12 (36.4)	< 0.0001
Number of papillary projection(s)‡				0.640
≤ 3	5/23 (21.7)	3/11 (27.3)	2/12 (16.7)	
> 3	18/23 (78.3)	8/11 (72.7)	10/12 (83.3)	
Flow in papillary projection(s)‡	19/24 (79.2)	8/12 (66.7)	11/12 (91.7)	0.630
Echogenicity of cystic fluid content				0.813
Anechoic	26/59 (44.1)	19/41 (46.3)	7/18 (38.9)	
Low‐level	28/59 (47.5)	18/41 (43.9)	10/18 (55.6)	
Ground glass	4/59 (6.8)	3/41 (7.3)	1/18 (5.6)	
Hemorrhagic	1/59 (1.7)	1/41 (2.4)	0/18 (0)	
Hyperechogenic foci	21 (13.7)	0/120 (0)	21 (63.6)	< 0.0001
Color score				0.004
1	1/150 (0.7)	1/117 (0.9)	0 (0)	
2	25/150 (16.7)	13/117 (11.1)	12 (36.4)	
3	66/150 (44.0)	52/117 (44.4)	14 (42.4)	
4	58/150 (38.7)	51/117 (43.6)	7 (21.2)	
Ovarian crescent sign	10/151 (6.6)	4/119 (3.4)	6/32 (18.8)	0.002
Acoustic shadows	9/152 (5.9)	6/119 (5.0)	3 (9.1)	0.383
Irregular margins	147 (96.1)	116 (96.7)	31 (93.9)	1
Ascites	57 (37.3)	45 (37.5)	12 (36.4)	0.328
Free fluid in PoD	76/152 (50.0)	63/119 (52.9)	13 (39.4)	0.168
Metastases	99 (64.7)	79 (65.8)	20 (60.6)	0.574

Data are presented as *n* (%), mean ± SD or *n*/*N* (%), where *N* is the number of participants with data available for each variable. Comparisons between HGSC and LGSC by two‐sided Pearson's chi‐square test or Fischer's exact test and Student's *t*‐test, as appropriate. *P* < 0.05 considered statistically significant.

* Data available for 147 patients.

† For multilocular and multilocular‐solid masses.

‡ If papillary projections present. PoD, pouch of Douglas.

### Radiomics analysis

#### Feature extraction

In total, 75 radiomics features were computed for each image. Features that did not produce a result were excluded, resulting in 66 radiomics features included in the analysis. Features having null or constant values across the different patients were also excluded, reducing the number to 58.

#### Feature selection and modeling

For the radiomics model, the feature selection process reduced the number of features from 58 to 14 by the univariate analysis and to 13 by the Boruta algorithm. We computed a correlation matrix to determine the pairwise association between features and remove redundancies (Figure 3). Ten features were removed because of high pairwise correlation, thus further reducing the number to three significant textural features: ‘gray‐level co‐occurrence matrix informational measure of correlation 2’ (‘F_cm.info.corr.2’) (*P* = 0.01), ‘gray‐level run length matrix run percentage’ (‘F_rlm.r.perc’) (*P* = 0.02) and ‘gray‐level size zone matrix zone size variance’ (‘F_szm.zs.var’) (*P* = 0.003). Only these three features were used for developing the radiomics model. The distribution of the three selected radiomics features that could differentiate between HGSC and LGSC is shown in Figure 4.

**Figure 3:**
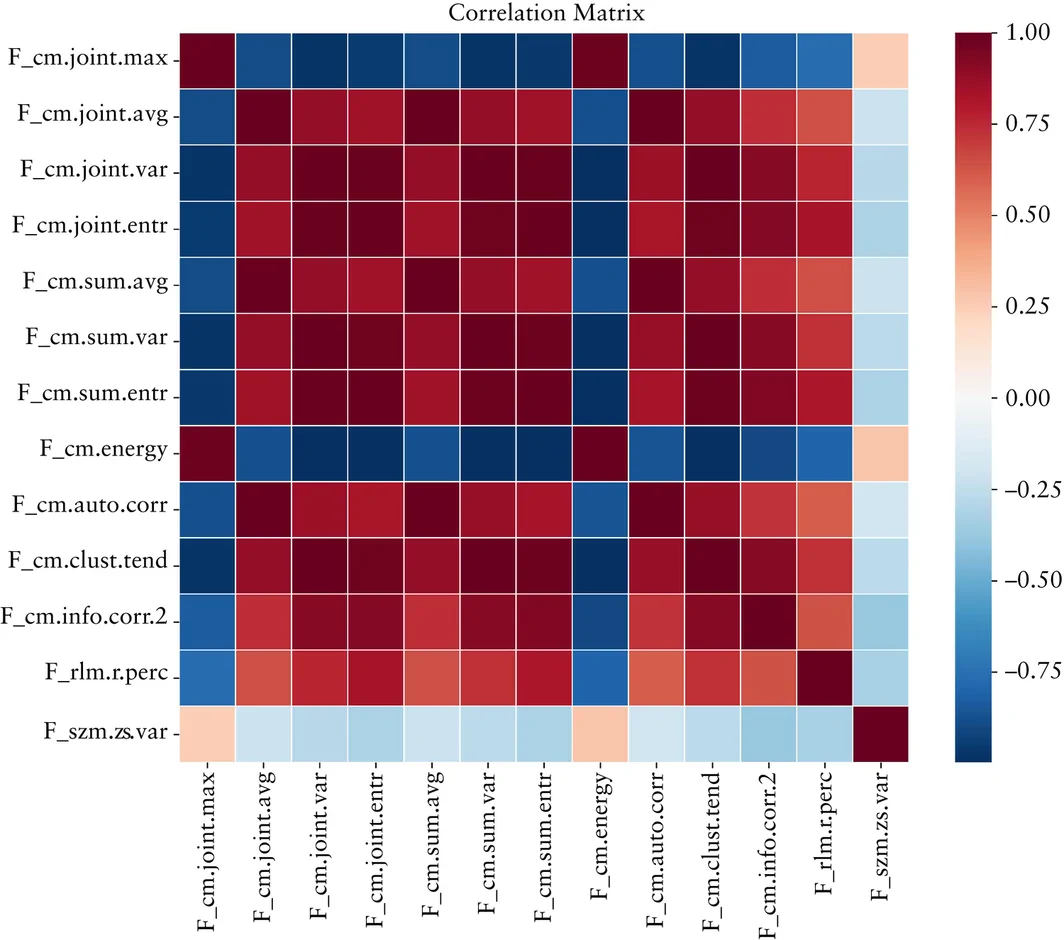
Correlation matrix to determine pairwise association between radiomics features in order to remove redundancies, showing linear correlation coefficients (*r*) computed for each pair of features, expressed via a colored scale: darker colors represent stronger correlations (*r* closer to ± 1) and lighter colors represent weaker correlations (*r* closer to 0).

**Figure 4:**
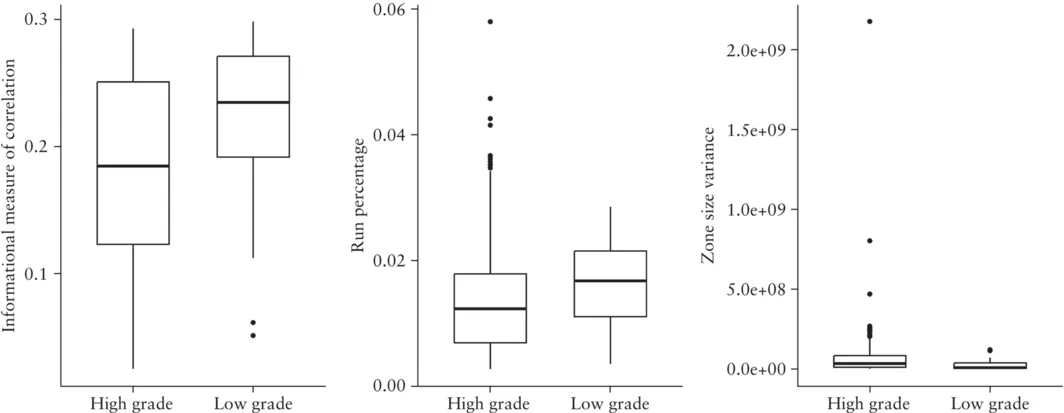
Box‐and‐whiskers plots of the features that showed statistically significant differences between high‐grade and low‐grade serous ovarian carcinomas. Boxes show median and interquartile range, whiskers are 1.5 × interquartile range, and circles indicate potential outliers.

For the clinical–radiomics model, only one radiomics feature (F_szm.zs.var) and age were selected after the feature selection process. Specifically, starting from 59 variables (including age), 15 features were retained after univariate analysis, 14 after application of the Boruta algorithm, and finally the number was reduced to three after Pearson correlation analysis with a threshold of 0.6. Following stepwise AIC regression, the final model included only two variables: F_szm.zs.var and age.

For the radiomics–ultrasound model, the same procedure as for the radiomics model was applied initially to the radiomics features. The three radiomics features that were selected for the radiomics model were then combined with three significant ultrasound variables identified via Fisher's exact test and the chi‐square test (color score, presence of papillary projection, presence of ovarian crescent sign). From the resulting six variables, one (F_szm.zs.var) was excluded owing to biserial correlation, and the final stepwise AIC regression selected two variables for the radiomics–ultrasound model: F_cm.info.corr.2 and color score.

The ultrasound model included three ultrasound features: color score, presence of papillary projections and presence of the ovarian crescent sign.

Details regarding model variables and their coefficients are provided in Tables S1–S4. The performance, expressed as ROC‐AUC, accuracy, sensitivity, specificity and PR‐AUC, of the radiomics, clinical–radiomics, radiomics–ultrasound and ultrasound models, both for model training and the optimism‐corrected performance metrics internally validated via bootstrap resampling, are reported in Table 3 and Figure S1. The clinical–radiomics model performed similarly to the ultrasound model (optimism‐corrected ROC‐AUC, 0.80 *vs* 0.78; *P* = 0.726) and better than the radiomics (optimism‐corrected ROC‐AUC, 0.80 *vs* 0.69; *P* = 0.042) and radiomics–ultrasound (optimism‐corrected ROC‐AUC, 0.80 *vs* 0.68; *P* = 0.048) models. The original PR‐AUC and the PR‐AUC bootstrap mean curves were consistently higher than the baseline across all models, indicating that each classifier outperformed random guessing (Figure 5).

**Table 3:** Discriminative performance of radiomics model, ultrasound model, radiomics–ultrasound model and clinical–radiomics model in prediction of high‐grade serous ovarian carcinomas (HGSC, *n* = 120) and low‐grade serous ovarian carcinomas (LGSC, *n* = 33)

	ROC‐AUC		Accuracy		Sensitivity		Specificity		PR‐AUC	
Model	Training	Bootstrap validtn.	Training	Bootstrap validtn.	Training	Bootstrap validtn.	Training	Bootstrap validtn.	Training	Bootstrap validtn.
Radiomics (threshold: 0.23)	0.72 (0.62–0.81)	0.69 (0.57–0.77)	0.66 (0.59–0.74)	0.64 (0.45–0.74)	0.82 (0.69–0.95)	0.78 (0.24–0.94)	0.62 (0.53–0.70)	0.60 (0.36–0.84)	0.38 (0.24–0.54)	0.34 (0.18–0.51)
Ultrasound (threshold: 0.27)	0.79 (0.68–0.88)	0.78 (0.68–0.88)	0.77 (0.74–0.84)	0.76 (0.69–0.84)	0.70 (0.54–0.85)	0.67 (0.33–0.83)	0.79 (0.72–0.86)	0.78 (0.70–0.95)	0.64 (0.47–0.77)	0.61 (0.45–0.76)
Radiomics–ultrasound (threshold: 0.19)	0.69 (0.59–0.78)	0.68 (0.56–0.77)	0.61 (0.53–0.69)	0.59 (0.40–0.71)	0.79 (0.65–0.93)	0.76 (0.30–0.95)	0.56 (0.47–0.65)	0.55 (0.30–0.78)	0.37 (0.23–0.51)	0.35 (0.18–0.48)
Clinical–radiomics (threshold: 0.32)	0.81 (0.72–0.89)	0.80 (0.72–0.90)	0.80 (0.73–0.86)	0.79 (0.69–0.84)	0.67 (0.51–0.83)	0.65 (0.29–0.80)	0.83 (0.77–0.90)	0.83 (0.68–0.95)	0.61 (0.44–0.75)	0.60 (0.45–0.77)

Data in parentheses are 95% CIs. Boostrap validtn., optimism‐corrected validation; PR‐AUC, area under the precision‐recall curve; ROC‐AUC, area under the receiver‐operating‐characteristics curve; Training, during model training.

**Figure 5:**
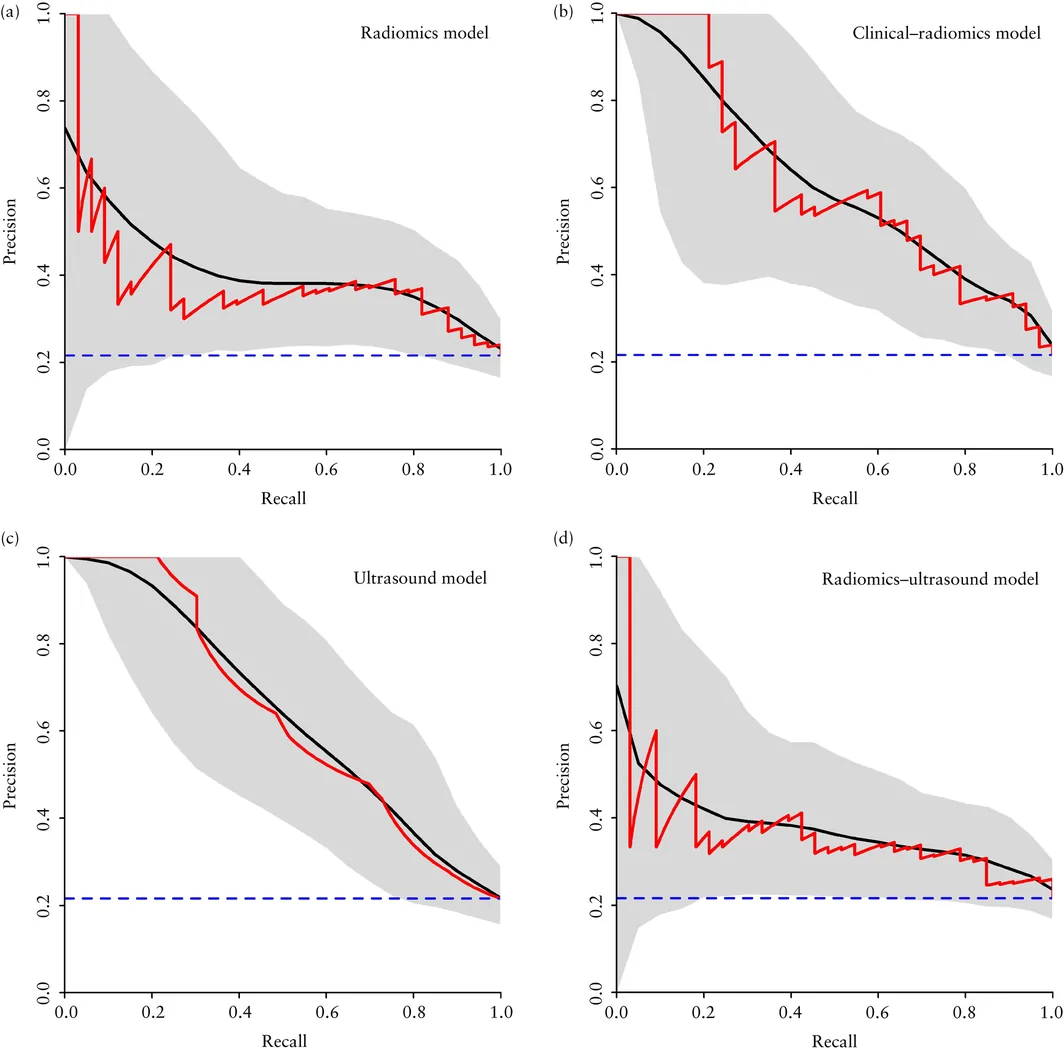
Precision‐recall (PR) curves for performance of radiomics (a), clinical–radiomics (b), ultrasound (c) and radiomics–ultrasound (d) models in differentiating between high‐grade and low‐grade serous ovarian carcinomas, during model training (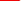, original PR curve) and internal validation (optimism‐corrected) (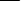, mean bootstrap PR curve), with 95% CIs around mean bootstrap PR curve (gray shading). The baseline (0.22) for this cohort is indicated (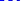); in all curves, area under the PR curve was higher than the baseline, indicating that the classifier consistently outperformed random guessing of predictions. Areas under the curve are presented in Table 3.

## DISCUSSION

In this study we have shown that radiomics features extracted from ultrasound images differ between HGSC and invasive LGSC. However, radiomics features did not provide additional information beyond that provided by conventional ultrasound. The clinical–radiomics model performed similarly to the ultrasound model and better than the radiomics and radiomics–ultrasound models.

Previous studies have focused on the preoperative diagnosis of serous tumors, aiming to identify ultrasound characteristics to distinguish HGSC from LGSC. Moro *et al*.^4^ described the ultrasound characteristics of 300 HGSC and 31 invasive LGSC from two institutions, one of which also contributed cases with tumors to this study. None of the cases in the current analysis was included in the previous study. The ultrasound features that they observed matched those observed in our series, with the exception of hyperechogenic foci: HGSC were generally multilocular‐solid (32.7% in their study *vs* 30.8% in the present study) or solid (64.0% *vs* 64.2%), with papillations in only a small percentage of cases (7.0% *vs* 10.0%). Similarly, invasive LGSC were multilocular‐solid (54.8% *vs* 42.4%) or solid (32.3% *vs* 45.5%), but with papillations in over one‐third of cases (37.5% *vs* 36.4%) and hyperechogenic foci, which were reported more frequently in our cohort (38.7% *vs* 63.6%). These findings are consistent with the histological characteristics reported in the 2014 World Health Organization (WHO) classification^31^, which describes both HGSC and LGSC as solid masses in most cases. However, in contrast to our findings, WHO reported bilateral adnexal masses in approximately 50% of cases, while bilateral involvement was observed in 21.7% of HGSC and 30.3% of LGSC in our cohort. This discrepancy may reflect differences in study populations, and the relatively small number of LGSC cases included in our analysis may limit the generalizability of our findings.

In recent years, several researchers have developed ultrasound‐based radiomics models for the classification of adnexal masses, mainly for discrimination between benign and malignant masses^32^, ^33^ and a few for the prediction of specific histology (for discrimination between Type‐I and Type‐II epithelial ovarian cancer^34^). In a retrospective study, Tang *et al*.^35^ extracted radiomics features from 206 lesions divided randomly into a training set (53 Type I + 71 Type II) and a test set (36 Type I + 46 Type II), and the seven features with the most differences were screened using the Lasso regression 10‐fold cross‐validation method. Their model including only radiomics features had similar performance to ours (ROC‐AUC, 0.73 *vs* 0.69), although their ROC‐AUC was obtained on a test set, whereas ours was derived from internal validation. Better performance than that of our model was found when radiomics features were extracted from other imaging modalities. Wang *et al*.^36^ developed a logistic‐regression model using radiomics features from computed tomography scans to differentiate HGSC from non‐HGSC, achieving a ROC‐AUC of 0.84 in the test cohort. The sample size in their study was larger than ours (665 patients *vs* 153 patients in our study). This may suggest that increasing the number of patients could improve the performance of the model.

Our findings suggest that preoperative discrimination between HGSC and LGSC is challenging, even with advanced radiomics approaches. To date, neither imaging‐ nor radiomics‐based models have achieved sufficient reliability to guide clinical decision‐making in the absence of histological confirmation. Any potential clinical application would require substantially better diagnostic performance, particularly in terms of improved sensitivity for HGSC. Moreover, the clinical utility of models aimed at distinguishing HGSC from LGSC is inherently limited by the rarity of LGSC, which account for less than 5% of epithelial ovarian cancer. Larger series and increased sample sizes could enable the development of new models with improved accuracy compared with that derived from this cohort. Nevertheless, we believe that this study represents a valuable preliminary step toward a comprehensive analysis of all ovarian histotypes. Such an approach, encompassing all histotypes, could pave the way for the future development of artificial intelligence (AI)‐based models capable not only of distinguishing between benign and malignant lesions, but also of predicting histotype and potentially integrating molecular analysis to support personalized patient counseling.

A major strength of our study is that all ultrasound examinations were performed by examiners with extensive experience and specific interest in gynecological‐oncology ultrasonography. The segmentation of all adnexal masses was performed manually by a EFSUMB Level‐II examiner. Another strength is that the radiomics analysis was conducted in accordance with the IBSI guidelines, ensuring methodological robustness and facilitating methodological comparability with other studies. The ultrasound images were obtained using different ultrasound systems, probes and scanning techniques, representing a ‘real‐world’ scenario, albeit the predominance of the GE Voluson E10 system may limit generalizability, as its image quality and device settings could have influenced the results.

Our study has several limitations, the most important being its retrospective nature and the small cohort of LGSC due to the low incidence of this histotype. The inclusion of only 33 cases of LGSC is insufficient for robust model training and validation, and raises concerns about generalizability. Moreover, the number of transabdominal images in our dataset was very small (10/332). The limited sample size precluded a separate, reliable analysis of this subgroup. The proposed clinical–radiomics model achieved a moderate optimism‐corrected ROC‐AUC (0.80). PR‐AUC was considered to address class imbalance and low disease prevalence. Manual segmentation of lesions may reduce the reproducibility of the study when performed by less experienced ultrasound examiners, and introduce operator‐dependent variability. Multiobserver segmentation could provide additional validation, but our approach prioritizes consistency and expertise, to ensure reliable and reproducible segmentations. The current limitations of ultrasound‐based radiomics involve the need for manual segmentation, which is time‐consuming. Semiautomatic or automatic software could improve this aspect and optimize radiomics pipeline reproducibility. We are currently developing a deep‐learning model for automated segmentation, to reduce the time and expertise required for manual segmentation and thereby improve the efficiency, consistency and feasibility of radiomics in clinical practice. Preprocessing images to remove speckle noise, and intensity normalization, could improve robustness, as texture features are sensitive to noise and ultrasound‐machine characteristics. Further studies are needed to understand the best methodology for radiomics analysis of ultrasound images.

Overall, our findings indicate that, despite detectable differences in radiomics features between HGSC and invasive LGSC, current radiomics and imaging‐based approaches do not provide sufficient added value over conventional ultrasound for reliable preoperative discrimination. Future research on larger, more comprehensive datasets integrating all ovarian histotypes and multimodal data may be necessary to develop more accurate and clinically useful AI‐based predictive models.

### COLLABORATORS

**F. Borella**, Division of Gynecology and Obstetrics 1, Department of Surgical Sciences, City of Health and Science University Hospital, University of Turin, Turin, Italy

**F. Buonomo**, Institute for Maternal and Child Health, IRCCS ‘Burlo Garofolo’, Trieste, Italy

**R. Fruscio**, Clinic of Obstetrics and Gynecology, Department of Medicine and Surgery, University of Milano‐Bicocca, Monza, Italy

**F. Pozzati**, Department of Woman and Child Health Fonazione Policlinico Universitario ‘A. Gemelli’ IRCCS, Rome, Italy

**R. Mancari**, Gynecologic Oncology Unit, IRCCS Regina Elena National Cancer Institute, Rome, Italy

[Correction added on 24 October 2026, after first online publication: The collaborator name M. Rosanna has been corrected to R. Mancari in this version.]

## Supporting information


**Tables S1–S4** Coefficients of radiomics model (Table S1), clinical–radiomics model (Table S2), ultrasound model (Table S3) and radiomics–ultrasound model (Table S4), including estimates, standard errors, *Z*‐values and *P*‐values.
**Figure S1** Receiver‐operating‐characteristics (ROC) curves for performance of radiomics, clinical–radiomics, ultrasound and radiomics–ultrasound models in differentiating between high‐grade and low‐grade serous ovarian carcinomas during model training (original ROC curve) and internal validation (optimism‐corrected; mean bootstrap ROC curve), with 95% confidence bands around mean bootstrap ROC curve.

## Data Availability

The data that support the findings of this study are available from the corresponding author upon reasonable request.
