# Supplementary material for: Differentiating high‐ and low‐grade serous ovarian carcinoma using radiomics: a pilot study
Source: Ultrasound Obstet Gynecol. 2026 Sep 13;68(4):559–70. doi: 10.1002/uog.70331 (PMC13641727; doi:10.1002/uog.70331)
Supplement: Supplementary file 1 [file UOG-68-559-s001.docx]

**SUPPORTING INFORMATION**

**Table S1** Coefficients of the radiomics model including estimates, standard errors, z-values and *P*-values

| **Variables** | **Estimate** | **Std. Error** | **z value** | **Pr(>\|z\|)** |
| --- | --- | --- | --- | --- |
| Intercept | 2.00 | 0.41 | 4.82 | 1.4e-06 |
| F_cm.info.corr.2 | -0.57 | 0.30 | 1.83 | 6.8e-02 |
| F_rlm.r.perc | 0.69 | 0.38 | 1.83 | 6.8e-02 |
| F_szm.zs.var | 3.90 | 1.71 | 2.28 | 2.3e-02 |

**Table S2** Coefficients of the clinical-radiomics model including estimates, standard errors, z-values and *P*-values

| **Variables** | **Estimate** | **Std. Error** | **z value** | **Pr(>\|z\|)** |
| --- | --- | --- | --- | --- |
| Intercept | 3.57 | 1.16 | 3.08 | 2.1e-03 |
| F_szm.zs.var | -2.65 | 1.24 | -2.13 | 3.3e-02 |
| Age | -0.10 | 0.02 | -4.46 | 8.38e-06 |

**Table S3** Coefficients of the ultrasound model including estimates, standard errors, z-values and *P*-values

| **Variables** | **Estimate** | **Std. Error** | **z value** | **Pr(>\|z\|)** |
| --- | --- | --- | --- | --- |
| Intercept | 1.19 | 0.96 | 1.24 | 2.1e-01 |
| Color score | -1.04 | 0.32 | -3.21 | 1.3e-03 |
| Ovarian crescent sign | 2.70 | 0.74 | 3.65 | 2.7e-04 |
| Papillary projection | 1.91 | 0.53 | 3.60 | 3.2e-04 |

**Table S4** Coefficients of the radiomics-ultrasound model including estimates, standard errors, z-values and *P*-values

| **Variables** | **Estimate** | **Std. Error** | **z value** | **Pr(>\|z\|)** |
| --- | --- | --- | --- | --- |
| Intercept | -3.67 | 1.06 | -3.45 | 5.6e-04 |
| F_cm.info.corr.2 | 0.57 | 0.22 | 2.51 | 1.2e-02 |
| Color score | 0.70 | 0.31 | 2.27 | 2.3e-02 |

**Figure S1** Receiver-operating-characteristics (ROC) curves for performance of radiomics, clinical–radiomics, ultrasound and radiomics–ultrasound models in differentiating between high-grade and low-grade serous ovarian carcinomas during model training and internal validation (optimism-corrected), with 95% confidence bands around mean bootstrap ROC curve.
